# 

**Published:** 1882-06

**Authors:** 


### Pamphlets.

---

True aneurism of the brachial artery at its upper third, cured by compression maintained for ten hours by means of a conical pad, with a resume of the literature of the subject. By L. Emmett Holt, A.M., M.D., New York. Reprint from the *American Journal of the Medical Sciences.*

Observations on hemiplegia, based on eighty-one recorded cases, with special reference to cerebral localization. By A. D. Rockwell, A.M., M.D., New York. Reprint from the *Medical Record.*

Our thirtieth year. Annual address before the Kentucky State Medical Society. Delivered April 5, 1882. By J. W. Holland, M.D., President. Louisville: J. P. Morton & Co. 1882.

The death-rate of Memphis. By George E. Waring, Jr., Newport. Reprint from the *American Architect.*

The opium habit; its successful treatment by the avena sativa. Read before the New Yark State Medical Society. By E. H. M. Sell, A.M., M.D. Reprint from the *Medical Gazette.*

Two cases of Malignant tumor of the sphenoidal cavities implicating vision. By Julian J. Chisolm, M.D., Baltimore. Reprint from the *Archives of Ophthalmology.*

Rupture of the eye-ball in the posterior hemisphere from a blow on on the face. By Julian J. Chisolm, M.D., Baltimore. Reprint from the *Archieves of Ophthalmology.*

Fourth annual report of the Board of Health of the city of Augusta, Ga.; Eugene Foster, M.D., President.

The mineral water controversy. Artificial and natural. Conflicting official opinions. The U. S. Chemist and the Attorney-General overruled by the Secretary of the Treasury. His novel chemical theory that natural products can be manufactured. A review of the whole question of American mineral waters, by Carl H. Schultz.

